# Oral Delivery of Curcumin Polymeric Nanoparticles Ameliorates CCl_4_-Induced Subacute Hepatotoxicity in Wistar Rats

**DOI:** 10.3390/polym10050541

**Published:** 2018-05-17

**Authors:** Gregory Marslin, Jose Prakash, Shanshan Qi, Gregory Franklin

**Affiliations:** 1 Ratnam Institute of Pharmacy and Research, Nellore 524 346, India; 2 College of Biological Science and Engineering, Shaanxi University of Technology, Hanzhong 723000, China; 3 Department of Pharmaceutics, Jaya College of Paramedical Sciences, Tiruninravur 602 024, India; jose88prakash@gmail.com; 4 Department of Pharmaceutics, Vels University, Chennai 600 117, India; 5 Dapartment of Pharmacology, Vitamin D research institute, Shaanxi University of Technology, Hanzhong 723000, China; qishanshan101@126.com; 6 Department of Integrative Plant Biology, Institute of Plant Genetics of the Polish Academy of Sciences, 34 Strzeszynska Street, PL-60-479 Poznan, Poland

**Keywords:** curcumin, polymeric nanoparticles, subacute hepatotoxicity, histopathology, behavioral changes, liver enzymes

## Abstract

Curcumin is the major bioactive compound of *Curcuma longa*, an important medicinal plant used in traditional herbal formulations since ancient times. In the present study, we report that curcumin nanoparticles (ηCur) protects Wistar rats against carbon tetrachloride (CCl_4_)-induced subacute hepatotoxicity. Nanoparticles of sizes less than 220 nm with spherical shape were prepared using PLGA and PVA respectively as polymer and stabilizer. Test animals were injected via intraperitoneal route with 1 mL/kg CCl_4_ (8% in olive oil) twice a week over a period of 8 weeks to induce hepatotoxicity. On the days following the CCl_4_ injection, test animals were orally administered with either curcumin or its equivalent dose of ηCur. Behavioural observation, biochemical analysis of serum and histopathological examination of liver of the experimental animals indicated that ηCur offer significantly higher hepatoprotection compared to curcumin.

## 1. Introduction

Liver is a vital organ of the body. As it plays important functions in digestion, metabolism and detoxification, which are necessary for survival, liver injury may lead to serious health consequences and even to death. Although treatment options are available, managing liver damage is still a challenge to the modern medicine. Polyherbal formulations are often prescribed as hepatoprotective agents [1], which generally contain a combination of natural antioxidants synergistically acting together to protect the liver cells from damage [2]. One such natural antioxidants is curcumin, which is extracted from *Curcuma longa*, an important medicinal plant used in traditional herbal formulations since ancient times [3,4].

Curcumin possesses anti-inflammatory, antimicrobial, antiviral, antirheumatic, and neuroprotective activities [5,6,7]. The excellent antioxidant property of curcumin has been the basis of its capacity to protect the liver against hepatotoxicity [8]. Curcumin has been shown to protect the liver against mercury-induced hepatic injuries and CCl_4_-induced liver fibrosis in rats [9,10]. The protective effect against liver fibrosis by curcumin is due to the inhibition of HIF-1α through an ERK-dependent pathway [10]. Curcumin also ameliorates CCl_4_-induced hepatic angiogenesis and sinusoidal capillarization by suppressing multiple proangiogenic factors [11].

In spite of these interesting bioactivities, the clinical applications of curcumin have been constrained by its hydrophobicity, poor bioavailability, and degradation at alkaline pH [12]. In order to overcome these limitations, nanoformulations of curcumin have been attempted. Although there are several reports available on curcumin nanoparticles targeting various diseases and ailments [13,14,15,16,17], only a few studies have been performed in the context of hepatoprotection. For instance, curcumin encapsulated in a tri block polymer (*N*-isopropylacrylamide, vinylpyrrolidone and acrylic acid) was tested for hepatoprotection in carbon tetrachloride (CCl_4_)-induced liver damage in an acute mice model [18]. Another short-term study compared curcumin loaded liposomes and curcumin loaded PLGA nanoparticles for hepatoprotection in Swiss albino rats [19].

In spite of the progress made in this line of research, curcumin nanoformulation has so far not been tested against subacute hepatotoxicity. Hence, the present study was conducted to evaluate the ability of curcumin nanoparticles to protect Wistar rats against CCl_4_-induced subacute liver damage.

## 2. Materials and Methods

### 2.1. Chemicals

Curcumin and PLGA (acid-terminated; lactide:glycolide 50:50; *M*w 24,000–38,000 D) and polyvinyl alcohol (PVA; *M*w 31,000–50,000 D) were purchased from Sigma-Aldrich Co. (St Louis, MO, USA). Carbon tetrachloride (CCl_4_) was purchased from Rankem Laboratory Reagent, New Delhi, India. All chemicals used in the present study were analytical grade.

### 2.2. Preparation of Curcumin Nanoparticles

Briefly, PLGA (100 mg) and curcumin (10 mg) were dissolved in acetone. This organic phase was added drop by drop into an aqueous solution containing 1% PVA and homogenized at 18,000 rpm (IKA T 25 Ultra turrax homogenizer, Staufen, Germany). The resulting emulsion was stirred for 4 h for the evaporation of the organic solvent and centrifuged at 47,000× *g* (Beckman Avanti J 25, Beckman Coulter Inc., Brea, CA, USA) for 15 min to obtain a pellet. After washing the pellet for three times in ultrapure water to remove any free drug and other excipients, it was freeze dried and stored at −20 °C as powder.

### 2.3. Characterization of Nanoparticles

The particle size and zeta potential of the formulations was determined by dynamic light scattering using a Zetasizer (Zetasizernano ZS, Malvern Instruments, Malvern, UK) [20]. All measurements were made at room temperature. Morphological characteristics of the ηCur were confirmed by transmission electron microscopy (TEM, JEM-1400, JEOL Ltd., Tokyo, Japan). Approximately 10 µL of nanoparticle suspension was placed in copper grid and allowed to air dry for 10 min. The samples were negatively stained with sodium phosphotungstate solution (2%, *w/w*) and visualized in TEM.

### 2.4. Encapsulation Efficiency

The encapsulation efficiency of the nanoformulation was determined as the percentage of curcumin entrapped in the nanoparticles. Briefly, 10 mg ηCur was dissolved in 1 mL of 90% methanol and sonicated for 5 min to disrupt the nanoparticles and to release the encapsulated curcumin. Resulting solution was centrifuged at 9391× *g* (Eppendorf 5424 R, Fisher Scientific, Hauppauge, NY, USA), and the supernatant was collected. Absorbance of the supernatant was read at 425 nm in a spectrophotometer (Shimadzu UV-1700, Kyoto, Japan) to quantify curcumin.

### 2.5. In vitro drug release

The in vitro release profile of curcumin from ηCur was tested by dialysis bag method. In brief, 10 mg of ηCur was suspended in 2 mL of phosphate-buffered saline (PBS) and transferred into a dialysis bag (molecular weight 12,000–14,000 Da). The bag was sealed with clips and suspended in 50 mL PBS pH 7.4 with 1% of Tween 80 under constant stirring at 100 rpm at 37 °C in a bottle [21]. At 1 h intervals, 1 mL PBS was withdrawn and the amount of curcumin was analyzed spectrophotometrically at 425 nm. To maintain sink conditions, 1 mL of fresh PBS was added to the bottle after each withdrawal.

### 2.6. Experimental Animals

Female Wistar rats weighing about 130–150 g were used for subacute toxicity studies. Permission for carrying out the experiment was obtained from the Animal Ethics Committee of the College of Pharmacy, Vels University, Chennai, India. Experimental animals were maintained at room temperature at 25 ± 2 °C with 12–12 h light-dark cycle and were fed with conventional laboratory diet and drinking water. All the protocols involving experimental animals were performed as per the Committee for the Purpose of Control and Supervision of Experiments on Animals (CPCSEA) guidelines.

### 2.7. Experimental Setup

A total of 36 animals divided randomly into six groups were used in the subacute study performed over a period of 8 weeks [22]. One group was administered with 1 mL/kg olive oil on Mondays and Thursdays repeatedly every week via intraperitoneal route and served as control. Rest of the five groups were intraperitonially administered with 1 mL/kg olive oil containing 8% CCl_4_ in the same manner to induce hepatotoxicity. Among them, one group served as CCl_4_ control (CCl_4_). Two groups were treated using orally administered 25 and 50 mg/kg free curcumin (Cur25 and Cur50) solubilized in normal saline with 100 µL of Tween 80, whereas two groups received 25 mg/kg (ηCur25) and 50 mg/kg (ηCur50) of curcumin equivalent nanoformulation on subsequent Tuesdays, Wednesdays, Fridays and Saturdays. Control and CCl_4_ groups received normal saline during these days. Throughout the study period, animals were observed for the development of toxicological signs such as mortality, change in body weight, feed intake etc., throughout the study period.

### 2.8. Biochemical and Histopathological Analysis of Liver

On the day 57, blood samples were collected from the retro-orbital plexus of each animal. The collected blood samples were allowed to clot and the serum was separated by centrifugation at 845× *g* (5424 R, Eppendorf, Hamburg, Germany) for 30 min at 4 °C. Aspartate transaminase (AST), alanine transaminase (ALT) and alkaline phosphatase (ALP) levels were analyzed in an autoanalyzer (Erba Smart Lab, Mumbai, India) using Erba test kits (Transasia, Mumbai, India). After collecting the blood samples, liver samples from the animals were collected and preserved in 10% formalin. Subsequently, the liver samples were fixed, embedded in paraffin using standard procedures and 5 µm thick sections were taken in a microtome (Leica RM 2235, Wetzlar, Germany). Tissue sections were mounted on glass slides, stained with hematoxylin-eosin and observed under a light microscope for photography. Photomicrographs were analyzed for any histopathological changes.

### 2.9. Statistical Analyses of Data

Data were analysed using GraphPad 5 software (Prism, San Diego, CA, USA). Data are expressed as the mean ± SD of at least three replicates. The statistical significance of means was evaluated using by Student’s *t*-test.

## 3. Results

### 3.1. Characteristic Features of Prepared Nanoparticles

**Figure 1:**
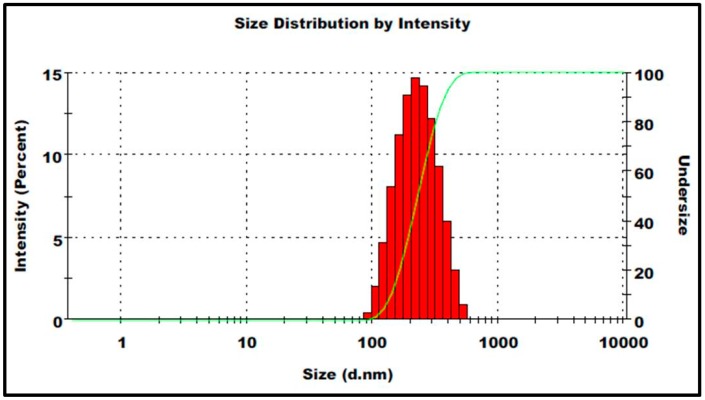
Histogram showing particles size and size distribution of ηCur.

**Figure 2:**
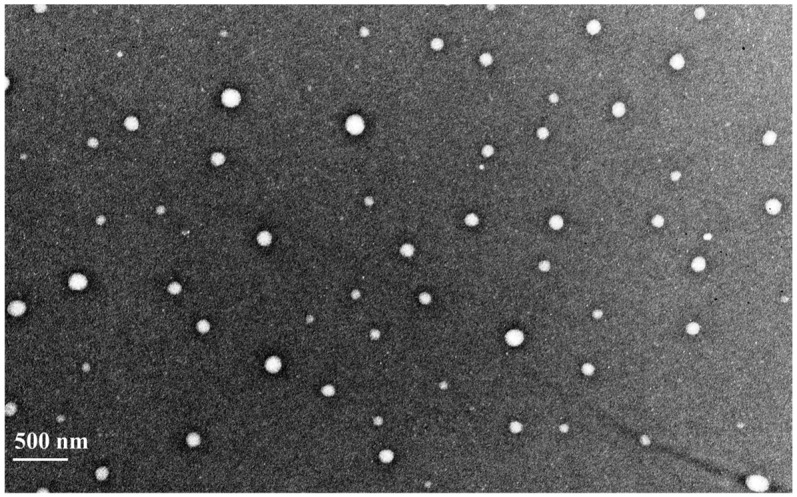
TEM image showing the spherical shape of ηCur.

The mean size of the prepared ηCur was 213 ± 3.6 nm as revealed by Malvern particle size analyzer (Figure 1). Correspondingly, most of the ηCur were less than 220 nm size, when analyzed by TEM (Figure 2). TEM images further revealed that the prepared ηCur possessed spherical shape without any aggregation. The zeta potential of ηCur was −26.6 ± 2.4 mV. The encapsulation efficiency of the nanoparticles was 69.80%.

### 3.2. Drug Release Profile

**Figure 3:**
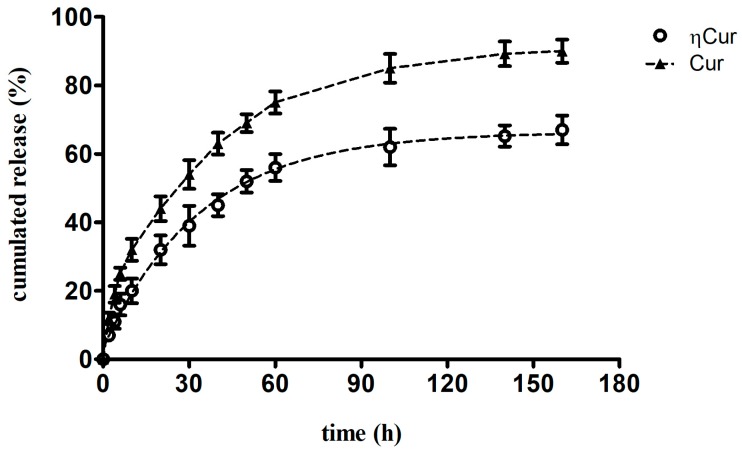
In vitro drug release profiles of curcumin nanoparticles (ηCur) and free curcumin (Cur) in PBS pH 7.4 and Tween 80. Values presented are mean ± SD of three replications.

Curcumin from the ηCur was released into the medium from the dialysis bag in a sustained manner for a period of 160 h after an initial rapid release for about 20 h (Figure 3). About 40% and 67% of curcumin from the ηCur was released at 30 and 160 h respectively. On the other hand, a relatively quick release of about 54% and 90% of curcumin at 30 and 160 h was observed for the free form of curcumin.

### 3.3. Behavioral Changes

The behavioral changes of animals observed during the study period are listed in Table 1. Animals injected with only CCl_4_ showed symptoms like diarrhea, sedation, weight loss, and reduced feed and water intake. Although animals treated with free curcumin did not suffer from diarrhea or sedation, their body weight, water and feed intake were reduced, irrespective of the dosage. Animals treated with both low and high ηCur dose did not show any of these symptoms.

### 3.4. Changes in Liver Damage Marker Enzymes

**Figure 4:**
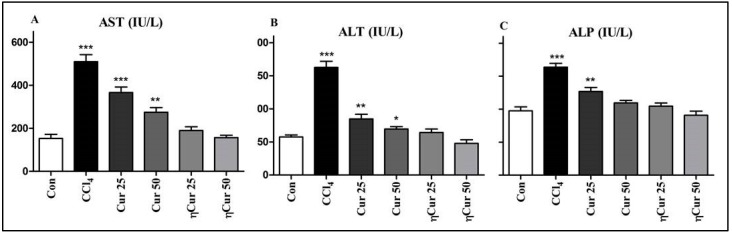
Changes in the level of liver damage marker enzymes in the serum. Graphs showing (**A**) aspartate transaminase (AST), (**B**) alanine transaminase (ALT) and (**C**) alkaline phosphatase (ALP) levels in control (Con) and experimental animals. CCl_4_—group of animals that received only carbon tetrachloride, Cur 25—group of animals orally administered with 25 mg/kg free curcumin after carbon tetrachloride injection, Cur 50—group of animals orally administered with 50 mg/kg free curcumin after carbon tetrachloride injection, ηCur 25—group of animals orally administered with 25 mg/kg curcumin in the form of nanoparticles after carbon tetrachloride injection, ηCur 50—group of animals orally administered with 50 mg/kg curcumin in the form of nanoparticles after carbon tetrachloride injection. Values marked with asterisks in a graph are significantly different from the respective control as analyzed by Student’s t-test (* *p* ˂ 0.05, ** *p* ˂ 0.01 and *** *p* ˂ 0.001). Data are presented as mean ± SD of three replications.

The highest level of AST, ALT and ALP was observed in the serum of animals which received only CCl_4_ (Figure 4). However, level of all these enzymes remained almost similar to that of control in the animals treated with ηCur 25, which is 25 mg/kg curcumin equivalent. When treated with 25 mg/kg free curcumin (Cur 25), the level of these enzymes was higher than the control. On the other hand, only the level of AST and ALT found elevated in the animals treated with a higher dose namely 50 mg/kg of free curcumin (Cur 50).

### 3.5. Histopathology of the Liver

**Figure 5:**
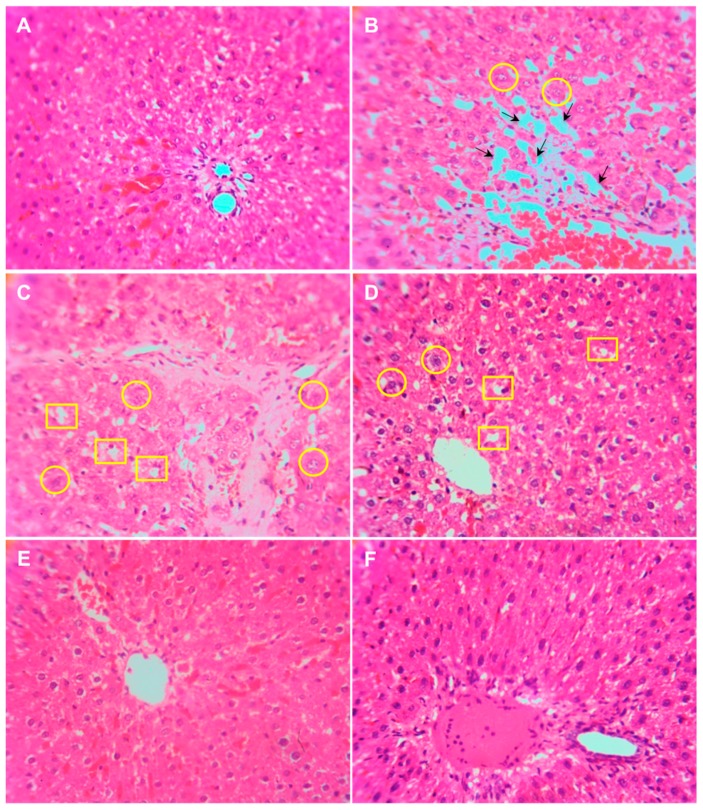
Photomicrographs showing normal architecture of hepatocytes in liver sample obtained from a control animal (**A**), disorganized hepatocytes, cellular inflammation, area necrosis, hepatic sinusoid and pathological nuclear cell division (circle) in animals injected with CCl_4_ (**B**), damaged hepatocytes, pathological nucleus cell division and steatosis in animals treated with 25 mg/kg of free curcumin (**C**), mild changes in the architecture, pathological nucleus cell division and mild steatosis in animals treated with 50 mg/kg of free curcumin (**D**) and normal hepatocytes architecture in animals treated with low and high doses of ηCur (**E**,**F**). Some of the areas showing hepatic sinusoid (arrow), pathological nuclear cell division (circle) and steatosis (square) are marked.

Histological examination of the liver samples from control animals revealed a normal hepatic architecture and polyhedral hepatocytes (Figure 5A). However, the liver of animals administered with CCl_4_ showed disorganized hepatocytes, multi focal area necrosis, congested blood vessel and fatty degradation (Figure 5B). Although the liver tissues of animals treated with free curcumin at both concentrations showed mild change in liver architecture and congested blood vessel (Figure 5C,D), no signs of damage was observed in liver tissue obtained from the animals treated with ηCur (Figure 5E,F).

## 4. Discussion

Pharmacological properties of *C. longa* are largely attributed to the antioxidant activity of curcumin, a major compound found in its rhizome (turmeric). Curcumin is widely known for its bioactivities against various ailments including hepatotoxicity. Although curcumin nanoformulations have been studied quite intensively for various purposes, we report on their ability to protect Wistar rats against CCl_4_-induced subacute hepatotoxicity for the first time.

Curcumin nanoparticles (ηCur) were successfully synthesized by emulsion solvent evaporation method. This method has been used to prepare stable nanoformulations of various therapeutic agents in other studies [23,24,25,26]. Since both polymer (PLGA) and the drug (curcumin) were dissolved in an organic solvent, mixing it with an aqueous phase containing stabilizer (PVA) under high-speed homogenization resulted in the formation of nano emulsion. Evaporation of the organic solvent from the emulsion results in the formation of ηCur stabilized with PVA. Nanoformulations prepared using PLGA generally possesses a biphasic drug release profile as observed in the present study, mainly due to the fact that the presence of 50% of glycolic acid in the polymer makes the resulting ηCur hydrophilic. Hence, initial water penetration into the matrix might be higher than the rate of polymer degradation resulting in bulk release of curcumin. The burst release of curcumin may also be attributed to the molecules adhering to the nanoparticle surfaces.

The results of subacute hepatotoxicity study presented here clearly demonstrate the potential benefits of ηCur against liver damage. CCl_4_ is a hepatotoxic agent widely used in preclinical animal studies [27]. Since administration of CCl_4_ can induce acute or chronic liver injury in rodents, it is extensively used as an experimental model [28]. When CCl_4_ enters into the animal body, it produces trichloromethyl free radicals and reactive oxygen species (ROS) after being metabolized by cytochrome P450 [29,30]. These metabolites initiate a lipid peroxidation chain reaction that leads to liver damage. Acute exposure to CCl_4_ can cause central nervous system depression and neurological symptoms, which may induce changes in physiological behavior [31]. Correspondingly, the animals administered with CCl_4_ showed symptoms such as diarrhea, sedation, weight loss and reduced feed and water intake in the present investigation. Although the subsequent treatment with curcumin could overcome CCl_4_-induced symptoms like diarrhea and sedation in these animals, they still showed changes in body weight, reduced water and feed intake. Nevertheless, the lack of such behavioral changes in animals treated with ηCur indicated that the nano form of curcumin offer better protection against subacute toxicity induced by CCl_4_.

Liver injury alters the membrane permeability and transport function of the liver, which leads to excessive leakage of liver enzymes such as AST, ALT and ALP that could be used as biomarkers of liver damage [32]. An increase in the level of these enzymes in both serum and liver tissues represent the extent of hepatocellular damage [12]. In the present study, AST, ALP and ALT levels were found elevated in animals administered with CCl_4_ demonstrating the incidence of liver damage in these animals. A reduction in the level of these enzymes compared to CCl_4_ administered animals was observed in animals treated with a low dose of free curcumin clearly show that curcumin has the potential to protect from liver damage. The enzymes level further lowered with a high dose of curcumin indicating that the hepatoprotection extended by curcumin is dose-dependent. Similarly, a dose-dependent but higher reduction reaching the similar level to control animals was observed in animals treated with ηCur. All these observations clearly show that curcumin could protect the animals from CCl_4_-induced liver damage only at higher dosage, whereas a similar protection could be achieved with a low concentration of ηCur. The ability of turmeric extract and curcumin to protect against liver injury by decreasing the activities of serum AST and ALT by improving the hepatic glutathione content, leading to a reduced level of lipid peroxidase has been reported [33]. Further, pre- and post-treatment of curcumin could normalize the increase in lipid peroxidation, and decrease in glutathione level, superoxide dismutase, catalase, glutathione-S-transferase, glutathione peroxidase, glutathione reductase, and NADPH quinone reductase activities observed in liver of rats exposed to lindane [34]. Correspondingly, curcumin loaded liposomes and PLGA nanoparticles provided a significantly higher hepatoprotection compared to curcumin against CCl_4_-induced liver damage in rats [19]. Similarly, administration of curcumin as nanoparticles showed better amelioration than free curcumin against arsenic-induced liver toxicity [35]. In the same manner, *Cuscuta chinensis* extract nanoparticles exhibited 5 times higher hepatoprotective and antioxidant activities compared to its equivalent concentration of *C. chinensis* ethanolic extract [36]. In addition, results of the above study demonstrated that the hepatoprotective and antioxidant effects of *C. chinensis* were higher in the nanoformulation than the ethanolic extract suggesting that the absorption and solubility of poorly soluble herbal medicines could be improved by delivering as nanoparticles [36].

The better protection offered by ηCur over free curcumin against the CCl_4_-induced subacute hepatotoxicity observed in the present study might be possibly attributed to the enhanced bioavailability of curcumin. This enhancement in the bioavailability might be due to the smaller size of ηCur, sustained release property and the role of P-glycoprotein (P-gp) efflux pump [37]. The nanoparticles of ηCur with a high surface area could have possibly enhanced the solubility and absorption of curcumin across the intestinal membranes. It is an established fact that a higher absorption window is available across the intestinal region compared to the stomach due to a higher residence of drugs in the former than the later. The sustained release pattern of curcumin from ηCur combined with an enhanced residence time in the intestinal tract could have enhanced the absorption of curcumin across the intestinal tract resulting in an enhanced bioavailability of curcumin. Additionally, as curcumin is a substrate for P-gp present in the intestine, its absorption is hindered [38]. However, PLGA present in ηCur could inhibit this P-gp flux. Thus, the ability of PLGA to inhibit P-gp flux could be another factor contributing to the enhanced bioavailability of curcumin in the current study.

Administration of rats with CCl_4_ caused extensive damage to the cellular architecture of liver, leading to centrizonal necrosis, portal fibrosis, and marked steatosis [19]. The pathological characteristics of liver of CCl_4_ administered animals closely resemble the structural changes of chronic hepatitis and cirrhosis in humans [22]. In the present study, histopathological examination of the liver samples collected from animals that were administered only with CCl_4_ showed the presence of disorganized hepatocytes, multi focal area necrosis, congested blood vessel and fatty degradation of the liver tissues confirming the induction of liver toxicity by CCl_4_. Liver sections of animals that were subsequently fed with free curcumin showed a mild change in liver architecture and congested blood vessel. However, there was no change observed in the liver architecture of animals treated with ηCur, which possessed normal hepatic architecture and polyhedral hepatocytes similar to the control arguing for its better efficacy compared to curcumin. Thus, the histopathological examination of liver from the test animals further endorsed the results of the biochemical studies. The beneficial effect of curcumin in protecting the liver architecture against CCl_4_-induced liver injury has already been reported [39,40]. Curcumin also significantly prevented the lindane-induced histological alterations the liver tissue of Wistar rats [34]. Interestingly, when the hepatoprotective effect of curcumin and equivalent concentration of curcumin nanoformulation was compared in rats administered with single dose of CCl_4_ (40% *v/v* in olive oil, 1 mL/kg body weight), liver damage was prevented by administration of liposomal and nanoparticulate form of curcumin [19]. Recently, redox-sensitive β-CD-based nanoparticles with controlled release improved the therapeutic effect of curcumin against liver cancer in vitro [41].

## 5. Conclusions

Data from the present subacute study clearly show that oral administration of ηCur attenuates CCl_4_-induced subacute liver injury in rats. Our results provide a valuable confirmation of the hepatoprotective effect of curcumin nanoformulations previously shown in acute models. The therapeutic effect shown by high dose of curcumin could be achieved by a relatively low dose of ηCur. The enhanced hepatoprotection by ηCur compared to curcumin is due to the encapsulation in nanoparticles and sustained release profile of curcumin, which encourages better circulation kinetics and its ability to target liver. Thus, ηCur could be considered as a promising therapeutic strategy against subacute liver toxicity. As the liver damage is connected to ROS production and redox imbalance, redox sensitive nanoformulations targeting liver could still improve the therapeutic potential of curcumin. However extensive studies in terms of chronic liver toxicity, pharmacokinetic and pharmacodynamic effects of ηCur are warranted.

## Figures and Tables

**Table 1:** Behavioral changes in experimental animals after treatment with ηCur and free curcumin.

Parameters	Control	CCl_4_	Cur 25	Cur 50	ηCur 25	ηCur 50
Convulsion	-	-	-	-	-	-
Salivation	-	-	-	-	-	-
Diarrhea	-	+	-	-	-	-
Mortality	-	-	-	-	-	-
Sedation	-	+	-	-	-	-
Skin irritation	-	-	-	-	-	-
CNS Depression	-	-	-	-	-	-
Body weight	-	+	+	+	-	-
Feed intake	-	+	-	-	-	-
Water intake	-	+	+	+	-	-

CCl_4_—group of animals that received only carbon tetrachloride; Cur 25—group of animals orally administered with 25 mg/kg free curcumin after carbon tetrachloride injection; Cur 50—group of animals orally administered with 50 mg/kg free curcumin after carbon tetrachloride injection; ηCur 25—group of animals orally administered with 25 mg/kg curcumin in the form of nanoparticles after carbon tetrachloride injection; ηCur 50—group of animals orally administered with 50 mg/kg curcumin in the form of nanoparticles after carbon tetrachloride injection; + = Change observed in the tested parameter; - = No change observed.
